# Investigating Possible Synergism in the Antioxidant and Antibacterial Actions of Honey and Propolis from the Greek Island of Samothrace through Their Combined Application

**DOI:** 10.3390/foods11142041

**Published:** 2022-07-10

**Authors:** Evdoxia Postali, Panagiota Peroukidou, Efstathios Giaouris, Alexandros Papachristoforou

**Affiliations:** 1 Laboratory of Food Microbiology and Hygiene, Department of Food Science and Nutrition, School of the Environment, University of the Aegean, Ierou Lochou 10 & Makrygianni, 81400 Myrina, Greece; fns17104@fns.aegean.gr (E.P.); fns16099@fns.aegean.gr (P.P.); alpapach@aegean.gr (A.P.); 2 Department of Food Science and Technology, School of Agriculture, Aristotle University of Thessaloniki, 54124 Thessaloniki, Greece

**Keywords:** honey, propolis, Samothrace, Greece, antioxidant action, antibacterial action, synergism, bacterial pathogens, functional foods, novel antimicrobials

## Abstract

Several honeybee products are known for their functional properties, including important antioxidant and antimicrobial actions. The present study examines the antioxidant activity (AA), total polyphenolic content (TPC), and antibacterial action of honey and propolis samples collected from the Greek island of Samothrace, which were applied in vitro either individually or in combination in selected concentrations. To accomplish this, the 2,2-diphenyl-1-picrylhydrazyl (DPPH) scavenging activity and the Folin–Ciocalteu assays were employed to determine the AA and TPC, respectively, while the antibacterial action was investigated against each one of four important pathogenic bacterial species causing foodborne diseases (i.e., *Salmonella enterica*, *Yersinia enterocolitica*, *Staphylococcus aureus*, and *Listeria monocytogenes*) using the agar well diffusion assay. Compared to honey, propolis presented significantly higher AA and TPC, while its combined application with honey (at ratios of 1:1, 3:1, and 1:3) did not increase these values. Concerning the antibacterial action, *Y. enterocolitica* was proven to be the most resistant of all the tested bacteria, with none of the samples being able to inhibit its growth. *S. enterica* was susceptible only to the honey samples, whereas *L. monocytogenes* only to the propolis samples. The growth of *S. aureus* was inhibited by both honey and propolis, with honey samples presenting significantly higher efficacy than those of propolis. Νo synergism in the antibacterial actions was observed against any of the tested pathogens. Results obtained increase our knowledge of some of the medicinal properties of honey and propolis and may contribute to their further exploitation for health promotion and/or food-related applications (e.g., as preservatives to delay the growth of pathogenic bacteria).

## 1. Introduction

Foodborne diseases are considered major causes of morbidity and mortality worldwide [1]. Alarmingly, many people still suffer and die from such otherwise preventable and curable diseases, mainly in developing countries because of the lack of good hygienic practices and basic health care [2]. The most common output of such diseases is acute gastroenteritis [3]. This consists of an important public health matter and refers to the intense clinical onset of gastrointestinal symptoms, which are not related to preexisting health conditions, the use of drugs, or other non-infectious causes [4]. Bacterial pathogens together with enteric viruses are the main causes of acute gastroenteritis, whose clinical appearance varies from mild to severe illnesses that can be fatal in some cases [5,6].

Bacteria are responsible for 10–55% of gastrointestinal infections globally, with instances dependent upon factors such as population demographics and geographical area; high rates also occur in the industrialized world [3]. Of all the types of gastroenteritis, bacterial gastroenteritis is considered as the most dangerous one because of the possible severity of the symptoms, which may include abdominal pain, bloody stools, and prolonged diarrhea [7]. *Salmonella enterica*, *Yersinia enterocolitica*, and *Listeria monocytogenes* are bacterial species that are involved in foodborne cases of gastroenteritis worldwide [8]. Based on the latest available data for Europe, there were 52,702, 5668, and 1876 confirmed cases of salmonellosis, yersiniosis, and listeriosis in 2020, with case fatality ratios (%) of 0.19, 0.07, and 13.0, respectively [9]. In that year, salmonellosis and yersiniosis were the second and third most commonly reported zoonoses for humans in Europe, exceeded only by campylobacteriosis. *Staphylococcus aureus* is another bacterial species that, besides being involved in a variety of minor to severe community and hospital-acquired infections [10], frequently causes food poisoning as it can produce several heat-stable enterotoxins during its growth in foods [11].

Antibiotics are administered to treat the most severe cases of bacterial infections, including foodborne ones. These stop the in vivo growth of the bacteria and may also cause their death [12]. However, antibiotics are concurrently associated with numerous side effects, including hypersensitivity, immunosuppression, and allergic reactions, and they can also lead to the depletion of beneficial microorganisms inhabiting the intestine and the mucosa, thereby increasing the chances of later colonization by pathogens [13,14]. Antimicrobial resistance against antibiotics is also a major global public health problem [15]. Thus, many of the clinically relevant bacteria are now resistant to several of the frequently administered antibiotics [16]. Therefore, novel alternative antimicrobials for the effective treatment of infectious diseases are required. On the other hand, in the food industry, safe, cost-effective, and preferably green antimicrobials are needed to delay microbial growth in foods. In this direction, the further exploitation of some natural products and/or the discovery of new ones is currently being investigated [17].

Honey is antimicrobial against many pathogens, including well-known bacterial enteropathogens such as *Salmonella* spp., Shiga toxin-producing *Escherichia coli* (STEC), and *Campylobacter* spp. [18,19]. For instance, honey has also been used to reduce the duration of vomiting and diarrhea in infants and children suffering from acute gastroenteritis [20]. Besides its antimicrobial potency, several, mostly in vitro studies have also indicated its anticancer and antidiabetic actions [21]. Its therapeutic effects are believed to have resulted from the presence of various antioxidant and antimicrobial molecules, including hydrogen peroxide and phenolic compounds such as flavonoids and phenolic acids [21,22].

Well-known in the fields of beekeeping and apitherapy, propolis is a natural multicomponent resinous substance produced by honeybees that forage blossoms and resins from the buds and secretions of various plant species. It is mixed with pollen and enzymes produced by bees and is used in building honeycombs and in sealing and sterilizing hives, thus protecting the colony from disease, invaders, and generally ensuring a clean environment [23]. This is achieved through its known antimicrobial properties [24,25]. At the same time, propolis is also known to present significant antioxidant action, which makes it a promising functional mixture for relevant prevention and treatment applications [26,27].

Studies on the use of natural products, such as honey and propolis, for antioxidant-related applications as well as to combat detrimental microorganisms are constantly increasing [28]. However, research on these two substances has until now tended to focus on them individually and independently of each other. Nevertheless, there are still some promising data on the synergistic biological effects of the combined use of these two hive products, either alone or together with other bioactive compounds (e.g., antibiotics) [29,30,31]. Such a combined action could further increase their functional properties and also lower their minimum effective concentrations. To this end, the present study examines the antioxidant and antibacterial activities of honey and propolis—both derived from Samothrace, an island in north-eastern Greece known for its unique floral biodiversity [32]—applied either individually or in combination.

## 2. Materials and Methods

### 2.1. Collection of Honey and Propolis Samples

The type of honey used was a blossom honey, harvested during spring and early summer of 2021 by beekeepers with apiaries on the island of Samothrace. Its main botanical source was the tree *Arbutus andrachne* L. (Ericaceae), commonly called the Greek strawberry tree, which is native to the Mediterranean region and the Middle East [33]. The botanical origin of the honey was verified through a melissopalynological analysis, conducted according to standard methods [34] and as previously described [18]. This specific type of honey was selected since it has been described as being highly antioxidant and very rich in TPC [35].

Propolis samples were collected from hives of *Apis mellifera macedonica* in the north-western area of the same island. The specific type of propolis examined was “red propolis”, described as being highly antioxidant [36]. Crude propolis samples were frozen at −18 °C and ground to a fine powder of 50–60 μm in a chilled grinder (Kenwood, KMM060). The propolis powder was then dissolved in an ethanolic extract of viniculture by-products (EEVBP) in order to produce a commercial-like, edible tincture [37] containing 30% (*w/v*) propolis and 31.5% (*v*/*v*) ethanol. This tincture was then double-filtered through a paper filter with a pore size of 8 μm to remove any impurities. The EEVBP used as a solvent was a distillate by-product of the production process of tsipouro, a Greek traditional alcoholic drink, which was kindly donated by a local producer in Samothrace. Both samples (honey and propolis tincture) were transported (within one week of their collection/preparation) in dark glass vessels to the laboratory and stored in the refrigerator until their analyses.

### 2.2. Bacterial Strains and Preparation of Their Working Suspensions

Four bacterial species (one strain/species) were used as the target microorganisms in the antimicrobial assays. Their codes, isolation origins, and some other relevant information are presented in Table 1. Two of them were Gram-positive (*S. aureus, L. monocytogenes*), whereas the other two were Gram-negative (*S. enterica*, *Y. enterocolitica*). Before their use in the assays, they were stored deep-frozen at −80 °C in a Brain Heart Infusion (BHI) broth (Lab M, Heywood, Lancashire, UK) containing 15% (*v*/*v*) glycerol. When needed for the experiments, each strain was resuscitated through its streaking on the surface of Tryptone Soy Agar (TSA; Lab M) and its incubation at 37 °C for 24 h (preculture). Working cultures were prepared by inoculating a discrete colony from each preculture into 10 mL of fresh Tryptone Soy Broth (TSB; Lab M) and then incubating at 37 °C for 24 h. The purity of each working culture was always confirmed through its streaking on TSA and incubation under the same conditions.

### 2.3. Chemicals

The 2,2-diphenyl-1-picrylhydrazyl (DPPH), Trolox^TM^ and gallic acid were obtained from Sigma-Aldrich Chemie GmbH (Taufkirchen, Germany). Methanol (MeOH), Folin–Ciocalteu reagent, and monohydrated sodium carbonate (Na_2_CO_3_) were obtained from Merck (Darmstradt, Germany).

### 2.4. Determination of the Antioxidant Activity (AA)

For the determination of the AA, the DPPH^•^ scavenging activity method was used, as previously described [41]. Before measurements, honey was diluted 1:1 (*v*/*v*) in distilled water, while the propolis tincture was diluted 1:200 (*v*/*v*) in pure methanol. In addition, the diluted honey (H) and propolis (P) samples were combined at the following ratios (*v*/*v*): 75:25 (PH 75-25), 50:50 (PH 50-50), and 25:75 (PH 25-75). For the assay, an aliquot of 0.025 mL of each diluted sample (individually or in combination) was added to 0.975 mL of the DPPH^•^ solution (100 μM in MeOH), thoroughly mixed with a vortexer (Scientific Industries Inc., Bohemia, NY, USA), and stored at rest, for 30 min in darkness. The absorbance of each solution was measured at 515 nm using a UV-Vis spectrophotometer (UV-1800, Shimadzu Co., Kyoto, Japan), at t = 0 and t = 30 min. Trolox^TM^ equivalents (μM TRE) were determined from linear regression after plotting the %ΔA_515_ of known solutions of Trolox^TM^ against their concentrations, where %ΔA_515_ = ((A^t=0^_515_ − A^t=30^_515_)/A^t=0^_515_) × 100. Results were expressed as μmol TRE/mL. For each sample, each absorbance measurement was repeated six times.

### 2.5. Determination of the Total Phenolic Content (TPC)

The TPC of each sample (prepared as described for the determination of the AA) was evaluated using the Folin–Ciocalteu method, as previously described [41]. Briefly, in 3.16 mL of distilled water, 40 μL of each diluted sample (individually or in combination) and 200 μL of the Folin–Ciocalteu reagent were mixed. After shaking and resting for 1 min, 600 μL of sodium carbonate (20% *w/v* in distilled water) was added, and the sample was vortexed and stored in darkness for 2 h. The absorbance of each solution was then measured at 750 nm with a UV-Vis spectrophotometer. Results were expressed as g gallic acid equivalents (GAE)/L. For each sample, the absorbance measurements were repeated six times.

### 2.6. Determination of Antibacterial Action

For the determination of the antibacterial action, the honey sample was diluted in sterile distilled water in the following concentrations (*v*/*v*): 75, 50, 25, 12.5, 6.25, 3.13, and 1.56%. The propolis tincture was tested undiluted (100% *v*/*v*) as well as diluted in the following concentrations (*v*/*v*): 90, 75, 60, 50, 40, and 25%, all prepared in sterile distilled water. The undiluted propolis tincture was also tested following a purification procedure consisting of the overnight storage of the tincture at −80 °C, centrifugation at 3340 g for 2.5 min, and supernatant collection [41]. The undiluted honey (H) and propolis tincture (P) were also combined at the following ratios (*v*/*v*): 50:50 (HP 50-50), 75:25 (HP 75-25), and 25:75 (HP 25-75). Following the preparation of the samples, the antibacterial action of each one was determined against each of the targeted bacterial species using an agar well diffusion assay, as previously described [18]. In brief, liquid (25 mL) soft TSA medium (i.e., TSB also containing 0.7% *w/v* agar) was initially inoculated with the target microorganism (ca. 10^6^ CFU/mL) and placed in a Petri dish (90 mm diameter) where it was left to solidify. Eight wells (each with a diameter of 5 mm) were then created in the solidified medium with the help of an inverted sterile Pasteur glass pipette. Each sample (40 μL) was then placed in duplicate in the wells prepared in soft TSA, and the dishes were left for 1 h at room temperature and finally placed at 37 °C for 48 h. Following incubation, the growth inhibition zones around each well were measured with a ruler. Kanamycin sulfate (50 μg/μL; BP906; Thermo Fisher Scientific, Waltham, MA, USA) was employed as a positive antimicrobial control. Two negative antimicrobial controls were also used: one was 100% (*v*/*v*) sterile distilled water, and the other was a 70% (*v*/*v*) EEVBP solution (for the latter, this was the maximum concentration contained in the sample of 100% (*v*/*v*) propolis tincture). Each experiment was repeated three times, starting from independently grown bacterial cultures.

### 2.7. Statistical Analyses

One-way analysis of variance (ANOVA) was applied to evaluate the AA and TPC results, and their means were compared with the Games–Howell and the Tukey’s Honest Significant Difference (HSD) post hoc tests for AA and TPC, respectively. One-way ANOVA, followed by Fisher’s Least Significant Difference (LSD) post hoc test for mean pairwise comparisons were applied to check for any significant differences between the inhibition zone diameters (mm) of the different samples in the antimicrobial assays. All differences are reported at a significance level of 0.05.

## 3. Results and Discussion

### 3.1. AA and TPC of Propolis and Honey Samples

**Figure 1:**
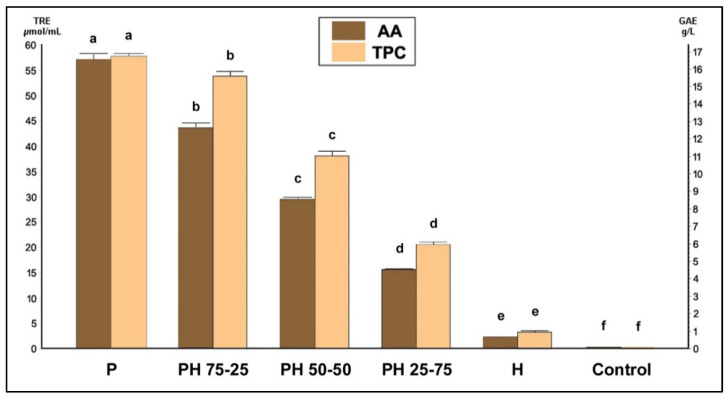
AA and TPC of the samples in TRE μmol/mL and g GAE/L, respectively. P: propolis tincture (100% *v*/*v*), H: honey (100% *v*/*v*), PH: propolis and honey mixtures at the ratios indicated. Control: EEVBP. Mean values followed by different superscript letters (a–f) significantly differ (*p* < 0.05).

Compared to the other samples, the pure propolis tincture presented the highest antioxidant values, with an average of 57.16 μmol TRE/mL (Figure 1), followed by the combined samples PH 75-25, PH 50-50, and PH 25-75 with average values of 43.64, 29.53, and 15.63 μmol TRE/mL, respectively. The honey sample showed the lowest AA, with 2.28 μmol TRE/mL. The antioxidant value of the EEVBP (used as the solvent to prepare the propolis tincture) was insignificant (0.16 μmol TRE/mL). Similar to the AA, the concentrations of TPC (Figure 1) varied between the samples, with the pure propolis tincture presenting the highest value of 16.72 g GAE/L, while the combined samples showed values of 15.64, 11.01, and 5.94 g of GAE/L for PH 75-25, PH 50-50, and PH 25-75, respectively. The TPC of honey was 0.94 g of GAE/L, while the TPC of the EEVBP was insignificant (0.019 g GAE/L).

Considering the net weight of the propolis tested in the assays (in g), the determined AA of its pure tincture (57.16 μmol TRE/mL) is equal to 922.3 μmol TRE/g, while its determined TPC (16.72 g GAE/L) is equal to 282 mg GAE/g. Results of the present study are thus in accordance with previous research, which revealed that Samothrace’s propolis presents a very high AA and is very rich in TPC [36,41]. Such functional properties are probably related to the island’s rich biodiversity [32]. Another study which compared the ethanolic extracts of propolis from various locations in Greece and Cyprus reported that the DPPH^•^ assay values ranged from 33 to 1110 μmol TRE/g, which were correlated with TPC values that ranged between 80.2 and 338.5 mg GAE/g [42]. A further study which analyzed the physicochemical characteristics of propolis collected from different locations in Greece found the highest polyphenolic content of 181 ± 7.8 mg GAE/g in the propolis of Imathia (region of Central Macedonia) [43]. It is thus clear that the propolis of Samothrace used in the current study presented higher AA and TPC than most of the samples mentioned in the studies above. Furthermore, its TPC is comparable to the highest reported values in the world [44,45,46,47].

Although the AA and TPC values of Samothrace honey (equal to 0.67 μmol TRE/g and 0.94 mg GAE/g, respectively) were lower when compared to propolis from the same island, the literature indicates that this honey can still be included amongst the top-rated honeys in Greece and in the world for these two attributes. Thus, the determination of the TPC in honey from Mount Olympus in Greece reported values of 0.55–0.92 mg GAE/g [48]. This older study also analyzed the TPC of manuka honey (a monofloral honey native to New Zealand and parts of Australia that has attracted great attention from researchers for its biological properties [49]) and found a value of 0.71 mg GAE/g [48]. A recent study which compared the TPC of Greek pine honeys from different locations reported values between 0.45 and 0.69 mg GAE/g [50]. Beyond Greece, a study conducted on Slovenian honeys showed that their polyphenolic content ranged from 0.02 to 0.23 mg GAE/g [51], while the phenolic concentration of Brazilian honeys ranged from 0.25 to 0.54 mg GAE/g [52].

According to the current results, it seems that there is a strong correlation between the AA and TPC of each sample, with the rate of propolis tincture mainly accounting for the total values of both these parameters in the case of the mixtures. Several previous studies on propolis and honey have also demonstrated a strong correlation between the AA and TPC of these products [46,48,53]. However, non-phenolic compounds such as diterpenes may also account for the AA of Samothrace’s red propolis [36].

### 3.2. Antibacterial Action of Honey and Propolis Samples

**Figure 2:**
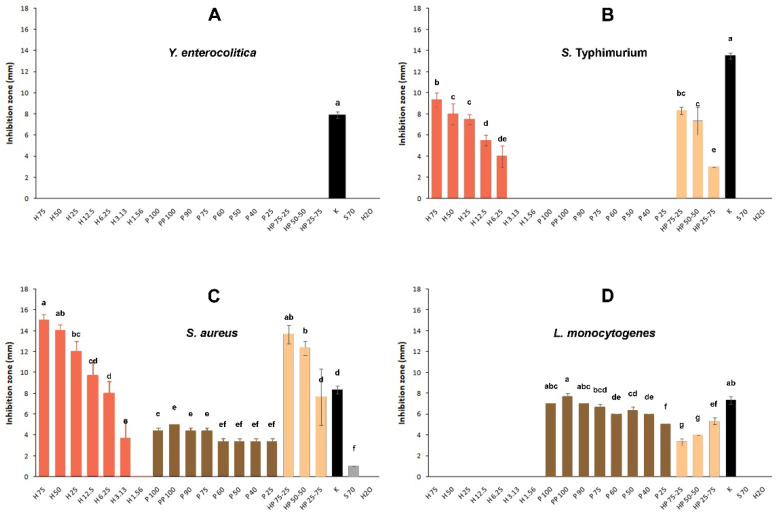
Inhibition zones (mm) of samples against the four bacterial species: (**A**) *Y. enterocolitica*; (**B**) *S. Typhimurium*; (**C**) *S. aureus*; (**D**) *L. monocytogenes*. H: honey (100% *v*/*v*), P: propolis tincture (100% *v*/*v*), PP: purified propolis tincture. Numbers indicate the concentration (% *v*/*v*) of each sample. HP: honey and propolis mixtures at the ratios indicated. K: Kanamycin, S70: Solvent (EEVBP) 70% (*v*/*v*), H_2_O: sterile distilled water. Mean values followed by different superscript letters (a–g) significantly differ (*p* < 0.05).

None of the tested samples presented antibacterial action against *Y. enterocolitica* (Figure 2A). Only kanamycin, a broad-spectrum antibiotic which was used here as the positive antimicrobial control, inhibited the growth of this bacterium by forming an inhibition zone of 7.89 ± 0.93 mm. *S. Typhimurium* was only found susceptible to honey samples when used at concentrations of ≥6.25% (*v*/*v*) (Figure 2B). Here, the sample containing 75% (*v*/*v*) honey was the most effective, presenting an inhibition zone of 9.33 ± 1.15 mm, with the antimicrobial effectiveness decreasing as the honey concentration decreased, and disappearing at a concentration of 3.13% (*v*/*v*) and below. All three mixtures (HP 75-25, HP 50-50, and HP 25-75) also presented antibacterial action, which seemed to be solely due to their honey content. Thus, the incorporation of propolis tincture does not seem to add to the antibacterial action already exhibited by the honey contained in each one of these mixtures.

All the tested samples, except honey applied at the lowest concentration of 1.56% (*v*/*v*), presented action against *S. aureus*, with the application of 75% (*v*/*v*) honey producing the maximum inhibition zone of 15.00 ± 1.00 mm (Figure 2C). It is worth noting that the inhibition provoked by the three other higher honey concentrations (50%, 25%, and 12.5% *v*/*v*) was significantly higher compared to that displayed by kanamycin (8.33 ± 1.12 mm). Regarding the propolis tincture, all its tested concentrations presented action against *S. aureus,* with no significant differences observed between them (inhibition zones ranged from 3.33 ± 0.58 to 5.00 ± 0.00 mm). As in the case of *S. Typhimurium*, all three mixtures (HP 75-25, HP 50-50, and HP 25-75) presented action against *S. aureus*, which again seemed to be solely due to their content in honey. No synergism in the antibacterial action upon the combined application of honey and propolis was observed.

The growth of *L. monocytogenes* was inhibited only by those samples containing the propolis tincture, either applied individually or in combination with honey (Figure 2D). In particular, the 100% (*v*/*v*) and 90% (*v*/*v*) propolis tinctures presented the strongest antibacterial action, which was equal to that displayed by kanamycin. The purification of the propolis tincture (through its overnight storage at −80 °C and centrifugation) did not alter its antibacterial action (against both Gram-positive bacteria). In the case of *L. monocytogenes*, the incorporation of honey in the honey–propolis mixtures (at 75, 50, and 25% ratios) seemed to decrease the antibacterial action of propolis since the inhibition zones displayed by the mixtures were always significantly lower than the respective samples containing only the propolis tincture (at 25, 50, and 75% ratios, respectively). At the same time, the sole application of honey against this pathogen, even at the maximum tested concentration (75% *v*/*v*), was always incapable of inhibiting its growth.

There has been much interest in the antimicrobial properties of honey in recent years [22,54]. Previous research shows that, depending on the mechanism of antimicrobial action, honeys can generally be divided into two groups: those with peroxide action and those with non-peroxide action. One of the most well-known non-peroxide honeys is manuka honey, whose antibacterial activity is related to the Unique Manuka Factor (UMF) rating, which is in turn correlated with the methylglyoxal and total phenol content [55]. Hydrogen peroxide (H_2_O_2_) is, on the other hand, the component mainly responsible for the antimicrobial activity of honeys with peroxide action. It acts as a strong oxidizing agent, forming free hydroxyl radicals that “attack” essential cellular components such as lipids, proteins, and DNA [56]. This is produced under aerobic conditions by glucose oxidase from glucose. Other factors that contribute to the antimicrobial activity of honey include high osmolarity, which lowers its water activity to values lower than 0.6 (due to the high amount of sugars; >80% *v*/*v*), high acidity (pH ca. 3.2–4.5), and its rich content of phenolic compounds, as well as other phytochemicals (such as flavonoids) and antimicrobial peptides (such as bee defensin-1) [22]. All these can inhibit bacterial growth, destroy bacterial cell structure, and ultimately cause bacterial death [54]. It is also known that the concentrations of the antimicrobial compounds contained in honey may vary depending on the source of the nectar (botanical origin), the geographical area and the local climatic environment, the species of honeybees, as well as the method of processing and the storage of honey [57].

In this study, honey was only effective against *S. Typhimurium* and *S. aureus*. The fact that *S. Typhimurium* is a Gram-negative bacterium might explain its inhibition, as Gram-negative bacteria are usually more susceptible than Gram-positive bacteria to various stresses, including an osmotic one caused by the high sugar content of honey, probably due to their thinner peptidoglycan layer. However, even though *S. aureus* is a Gram-positive bacterium, the tested honey had a strong effect on its growth. The sensitivity of this pathogen towards H_2_O_2_ [58] may account for its high inhibition, but further research on the antibacterial mechanisms of the specific honey tested here is needed. However, the complexity and variability of honey compositions present a great challenge. To facilitate our understanding on the antibacterial mechanisms of honeys, some simplified model systems have been successfully employed [59].

The propolis tincture was found to be effective only against the two Gram-positive bacterial species (i.e., *S. aureus* and *L. monocytogenes*). This seems to be in accordance with previous research [47,60]. The antimicrobial properties of propolis are known to be mainly attributable to its flavonoids, but also to other components such as caffeic acids and phenolic esters, which may act synergistically with each other [25]. In addition, the ability of propolis to affect the cytoplasmic membrane and the enzymatic activity of bacteria may also be responsible for the growth inhibition effects (especially against Gram-positive bacteria). On the other hand, in the current study, *Y. enterocolitica* was found to be an extremely resistant bacterium, with none of the samples being able to inhibit its growth. Alarmingly, this pathogen can also carry multi-antibiotic and metal-resistant determinants, which is a result of horizontal gene transfer in the environment, agriculture, and animal husbandry [61]. Additional studies should try to shed light on the cellular and molecular mechanisms that may be responsible for the extreme resistance of this bacterium to both honey and propolis, applied either individually or in combination.

### 3.3. Combined Application of Honey and Propolis

In studying the combined application of both honey and propolis at some selected ratios (i.e., 1:1, 3:1, and 1:3), no synergism was observed in either the antioxidant or antibacterial actions; however, surprisingly, an antagonistic activity was observed when these two products were applied together against *L. monocytogenes*. The lack of any obvious enhancement in the antimicrobial action against either *S. Typhimurium* or *S. aureus* may be due to the already existing strong action of honey when applied alone, masking any additional inhibitory effects of propolis when both were applied together. However, in the case of *L. monocytogenes*, honey was totally ineffective when applied alone, while it seemed to decrease the antibacterial action of propolis when these were applied together. This lack of antibacterial action from honey against this pathogen is in accordance with some previous results obtained by our team, where nine different honeys, including manuka, had been tested against *L. monocytogenes;* none was able to inhibit its growth [18].

According to previous research [31,62], the combination of different honeys with propolis extracts from Spain and Poland resulted in the degradation of the sensory properties of the mixtures, but also showed a noteworthy increase in their polyphenolic content and antioxidant activity, with the antimicrobial activity also increasing in some mixtures. In another recent study, propolis and honey mixtures were also found to display a stronger antibacterial effect than the activity exhibited by each individual sample, whereas the high antioxidant capacity of propolis was not affected when used in parallel with honey [30]. In another study which concurrently employed starch and honey [63], the addition of starch to five different honey samples increased their antimicrobial properties against *Pseudomonas aeruginosa*, an opportunistic and pathogenic bacterium well-known for its multidrug resistance and association with some serious hospital-acquired infections [64]. Studies have also investigated the antioxidant and/or antimicrobial actions combining honey with other honeybee products, such us bee bread and royal jelly. In such a study, the addition of both propolis and bee bread into honey resulted in the enhancement of its free radical scavenging ability, probably through the interactions occurring between the honeybee products; at the same time, this resulted in a strong antibacterial activity, especially against *E. coli* [65]. In another study, the addition of royal jelly in honey was found to improve its antibacterial effect against *S. aureus* [66].

## 4. Conclusions

Both honey and propolis from Samothrace, a Greek island known for its very rich plant biodiversity, were found to present strong AA and high TPC, confirming some previous relevant results. Regarding the antibacterial action, this was found to depend both on the honeybee product and the targeted bacterial species. Thus, honey was effective against *S. Typhimurium* and *S. aureus*, but not against *L. monocytogenes*. The latter bacterium was still susceptible to the action of propolis, which was also effective against *S. aureus*. Surprisingly, none of the tested samples could inhibit the growth of *Y. enterocolitica*, an emerging pathogenic bacterium provoking many cases of foodborne infections in Europe as well as in other countries in the last years. The combined application of both honey and propolis at three ratios (1:1, 3:1, and 1:3) did not seem to further increase the AA, TPC, and antibacterial action of the samples with respect to their individual application, whereas in the case of the mixtures, some antagonistic effects were observed against *L. monocytogenes*. Altogether, the results presented in this article increase our knowledge of some of the medicinal properties of honey and propolis and may contribute to their further exploitation for health promotion and/or food-related applications (e.g., as preservatives delaying the growth pathogenic bacteria).

## Figures and Tables

**Table 1:** Bacterial strains used in this study and relevant data.

Bacterial Species	Gram Reaction	Strain Code	Isolation Origin	Other Strain Information	Reference
*Staphylococcus aureus*	*+*	DFSN ^1^_B37	Greek traditional cheese	−	*unpublished*
*Listeria monocytogenes*	*+*	AAL ^2^ 20107	Mixed green salad	serovar 1/2b	[38]
*Salmonellla enterica*	−	FMCC ^3^_B137	Human salmonellosis outbreak	serovar Typhimurium, phage type DT193	[39]
*Yersinia enterocolitica*	−	DSM ^4^ 4780	Glanders-like infection of face	subsp. *enterocolitica*, type strain	[40]

^1^ Department of Food Science and Nutrition, Culture Collection of the Laboratory of Food Microbiology and Hygiene, University of the Aegean in Myrina, Lemnos, Greece; ^2^ Athens Analysis Laboratories SA, Microbiology Laboratory in Metamorfosi, Greece; ^3^ Food Microbiology Culture Collection, Laboratory of Microbiology and Biotechnology of Foods, Department of Food Science and Human Nutrition, Agricultural University of Athens in Athens, Greece; ^4^ DSMZ-German Collection of Microorganisms and Cell Cultures GmbH, Leibniz Institute in Braunschweig, Germany.

## Data Availability

The data presented in this study are available on request from the corresponding author.
